# Development and Application of Genetic Ancestry Reconstruction Methods to Study Diversity of Patient-Derived Models in the NCI PDXNet Consortium

**DOI:** 10.1158/2767-9764.CRC-23-0417

**Published:** 2024-08-16

**Authors:** Paul C. Lott, Katherine Chiu, Juanita Elizabeth Quino, April Pangia Vang, Michael W. Lloyd, Anuj Srivastava, Jeffrey H. Chuang, for the PDXNet Consortium, Luis G. Carvajal-Carmona

**Affiliations:** 1 The Health Equity Leadership, Science, and Community Research Laboratory, Genome Center, University of California, Davis, California.; 2 The Jackson Laboratory for Mammalian Genetics, Bar Harbor, Maine.; 3 The Jackson Laboratory for Genomic Medicine, Farmington, Connecticut.; 4 Department of Biochemistry and Molecular Medicine, School of Medicine, University of California, Davis, California.

## Abstract

Precision medicine holds great promise for improving cancer outcomes. Yet, there are large inequities in the demographics of patients from whom genomic data and models, including patient-derived xenografts (PDX), are developed and for whom treatments are optimized. In this study, we developed a genetic ancestry pipeline for the Cancer Genomics Cloud, which we used to assess the diversity of models currently available in the National Cancer Institute–supported PDX Development and Trial Centers Research Network (PDXNet). We showed that there is an under-representation of models derived from patients of non-European ancestry, consistent with other cancer model resources. We discussed these findings in the context of disparities in cancer incidence and outcomes among demographic groups in the US, as well as power analyses for biomarker discovery, to highlight the immediate need for developing models from minority populations to address cancer health equity in precision medicine. Our analyses identified key priority disparity-associated cancer types for which new models should be developed.

**Significance:**

Understanding whether and how tumor genetic factors drive differences in outcomes among U.S. minority groups is critical to addressing cancer health disparities. Our findings suggest that many additional models will be necessary to understand the genome-driven sources of these disparities.

## Introduction

Advances in our understanding of cancer genetics have led to the proliferation of improved precision treatments (1), yet there are disparities in the groups for which these treatments are most impactful. A major contributor to these disparities is an under-representation of donors with non-European ancestry in cancer cell lines, sequence data, and patient-derived models (2). Cancer health disparities are pervasive in US ethnic/racial minority communities and are driven by complex interactions between socioeconomic factors and potentially also by somatic and germline genetic variants differing in frequency between populations (3–5). Understanding whether and how genetic variants influence cancer health disparities will require a greater investment in developing models that better represent human genetic and epigenetic variation.

Genetic ancestry, which describes the relationships between individuals and populations based on shared genetic history, can provide insights into disease risk, prognosis, and therapy response. For example, studies in Latinos, who trace their ancestry to Europeans, African slaves, and Indigenous Americans, have shown that Indigenous ancestry has an inverse relationship with breast cancer risk (6, 7). This is consistent with data suggesting a low incidence of breast cancer in Latin American countries with large Indigenous American populations (8). Interestingly, we showed that Indigenous American ancestry is associated with breast tumor *ERBB2* amplification (9), while other studies have shown similar associations between Indigenous ancestry and *EGFR*-mutated lung tumors (10). Studies in African Americans (AA) have also shown that African ancestry is associated with a higher risk of prostate cancer (11), and that genetic variants exclusively found in Africa are associated with the prevalence of triple-negative breast tumors in AA patients (12). Despite these associations between genetic ancestry and cancer phenotypes, a major limitation in the field has been the paucity of germline and somatic data from patients from diverse populations (13). Another major limitation has been the lack of minority patient-derived models. Such patient-derived models recapitulate their genome and epigenome and are needed to advance precision health equity in such populations (2). To address these research gaps, the National Cancer Institute (NCI) has supported two centers within the patient-derived xenograft (PDX) Development and Trial Centers Research Network (PDXNet) to work with minority populations to create models reflecting their genetic background and exposures, to characterize their genome, and to facilitate studies leading to minority-focused clinical trials that address cancer health disparities.

In this manuscript, we describe the development of an ancestry estimation pipeline on the Seven Bridges Genomics (SBG) Cancer Genomics Cloud (CGC) as part of the NCI-PDXNet project. We use this new pipeline to describe the diversity of samples currently in PDXNet in terms of the genetic ancestry of patients from whom the tumors were sampled. We further present power analyses to argue that additional PDX models are critically needed to address the cancer health disparities in U.S. minority groups.

## Materials and Methods

Full method details are provided in the Supplementary Material. All patients who donated biospecimens for PDX model generation in PDXnet provided written informed consent. They were recruited using research protocols adhering to the Common Rule that were approved by IRBs at their corresponding institutions. In brief, we aggregated reference data from the 1,000 Genomes Project Phase III (14), GenomeAsia 100 K (15), and INMEGEN (16), filtered by minor allele frequency, Hardy-Weinberg equilibrium, linkage disequilibrium, and relatedness among individuals. We then used a principal component analysis to identify individuals with little admixture based on the continental ancestry group clustering. The result was a dataset of 264,153 SNPs from 1,990 individuals (Supplementary Table S1), which we used to prepare the weighting factors for ancestry inference with the program SNPweights v2.1 (17). We benchmarked the genetic ancestry estimates provided by this new reference panel using ADMIXTURE analyses. Finally, we developed an ancestry estimation workflow on the Cancer Genomics Cloud, which we used to quantify the diversity of PDX models in the PDXNet resource (Supplementary Table S2). To validate our ancestry estimation method and panel, we cross-validated our approach in a set of 1,000 Genome (14) individuals not used in the reference panel generation (Supplementary Data). The SNPweights panel estimates showed small differences for minority individuals compared to 1,000 Genomes (Supplementary Data; Supplementary Tables S3–S5). The largest difference was in Europeans, where the SNPweights panel showed a mean difference of −0.071 (SD: 0.023) compared to 1,000 Genomes estimates (Supplementary Table S5). The average differences of admixed AFR, AMR, EAS, and SAS individuals ranged from 0.000 (SD: 0.001) to 0.022 (SD: 0.040).

To contextualize the distribution of PDXnet genetic diversity we used epidemiological data from NCI to identify the top cancer outcome disparities for AAs and Latin Americans in the US (18, 19). We tabulated the number of relevant models currently available for understanding those disparities.

### Data availability

The ancestry estimation models were trained using three publicly available datasets: (i) 1,000 Genomes Phase III (https://www.internationalgenome.org/); (ii) INMEGEN (http://www.inmegen.gob.mx); and (iii) GenomeAsia 100 K (https://browser.genomeasia100k.org/). The datasets supporting the findings of this study are available from multiple centers and repositories. The three main repositories for these PDX datasets are: (i) Baylor College of Medicine (BCM) PDX Portal (https://pdxportal.research.bcm.edu/), (ii) NCI Patient-Derived Models Repository (PDMR, https://pdxportal.research.bcm.edu/), and (iii) Cancer Data Service (CDS, https://dataservice.datacommons.cancer.gov/). The PIs listed in these repositories can be contacted directly with data access requests. Data contributors and their respective repositories are as follows: BCM, Fred Hutchinson Cancer Research Center (PDMR), Huntsman Cancer Institute: HCI (CDS), Jackson Laboratory (PDMR), Mayo Clinic (PDMR), MD Anderson Cancer Center (PDMR), National Cancer Institute (PDMR), UC Davis , University of Alabama (PDMR), Wistar Institute (CDS), and Washington University in St. Louis: WUSTL (CDS). Some of the PDX Sequence Datasets used in the study include: (i) BCM/HCI, available from NCBI SRA, https://www.ncbi.nlm.nih.gov/bioproject/PRJNA756268); (ii) WUSTL, available from CDS (https://dataservice.datacommons.cancer.gov/#/study/phs002305); and (iii) Wistar Institute, available from CDS (https://dataservice.datacommons.cancer.gov/#/study/phs002432). Many of the PDXNet projects are ongoing, and dataset submissions to their respective repositories are in progress, but access to these datasets can be obtained upon request. Please contact the corresponding author, PDXNet, or the specific repository to obtain access to these datasets.

## Results

### Genetic ancestry among PDX models in PDXNet

After filtering reference data, the first three principal components explained 44.63%, 24.71%, and 17.6% of the variation of the filtered non-admixed ancestral reference genotype matrix, respectively. Samples clustered well by continental ancestry category among these three principal components (Supplementary Fig. S1), except for the Indigenous American and South Asian ancestry categories, which showed looser clustering, potentially due to population diversity or a lack of genome-wide references, particularly for Indigenous American populations.

**Figure 1:**
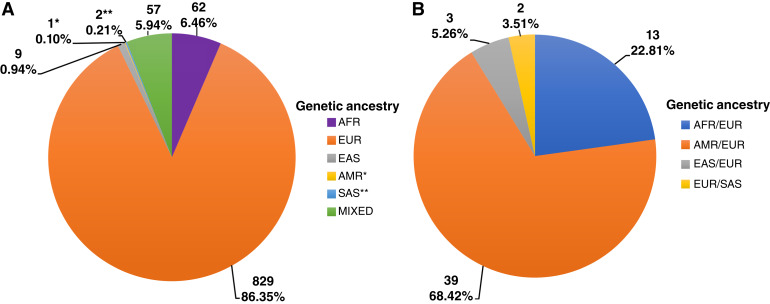
Diversity of genetic ancestry estimates from PDXNet models. **A,** Inferred genetic ancestry for 960 models across all cancer types. **B,** Top two categories for 57 “MIXED” samples with no category >70%. Numbers and percentages in each box reflect the models assigned to that category based on estimates from the SNPweights analysis.

The models available in PDXNet as of September 2022 represented 960 unique patients, with 606 having self-reported race and ethnicity information. These models were developed by the six PDXnet centers and the NCI PDMR. Our genetic ancestry pipeline estimated 62 models with majority African ancestry and one model with majority Indigenous American ancestry (Fig. 1A). Thirteen models had mixed African and European ancestry and 39 models had mixed Indigenous American and European ancestry (Fig. 1B). The estimates of genetic ancestry proportions were highly concordant with self-reported race and ethnicity information (Supplementary Table S2).

Overall, most of the models in PDXNet originate from patients with a predominant European ancestry (Fig. 1). Certain cancer types, such as breast cancers (Supplementary Fig. S2A and S2B), have a greater representation of patients from non-European backgrounds due to the efforts of minority PDXNet centers at Baylor College of Medicine and University of California at Davis.

### Power to detect drivers and develop new PDX models

In addition to estimating the PDX model genetic ancestry, we were interested in assessing whether their numbers were sufficiently large that we might discover alterations that were rare and potentially ethnicity- or race-specific. To do so, we carried out power analyses, which demonstrated that a study with 150 to 300 models in each of the two ancestry categories would have >80% to detect the presence of a driver mutation segregating between them (Supplementary Fig. S3). We found that for a driver mutation segregating at frequencies of 0.01 to 0.1 in a given population, sampling 50 to 100 individuals from that population should be sufficient to identify at least five patients from which PDX models could be developed (Supplementary Fig. S4). Although, to our knowledge, there are no guidelines or recommendations for the number of different models that should be used to obtain sufficiently robust preclinical data required for translation to clinical trials, our power estimation indicates that currently available models are insufficient for the study of rare and moderate frequency driver mutations.

### Model race/ethnicity and cancer health disparities

After completing our genetic ancestry analyses, we wanted to assess whether the available minority models were appropriate to address existing cancer health mortality disparities in the US. Our goal was, first, to identify which cancer types resulted in disproportionally higher rates (we called these “priority cancer health disparity malignancies”) in minority groups and second, we used self-reported donor race and ethnicity data from PDXNet models to estimate the number of race/ethnicity-appropriate models for each one of the priority cancer health disparity malignancies. To identify the priority cancer health disparity malignancies for AAs and Latinos, we used SEER data to list the top 10 causes of cancer mortality for Non-Latino Whites (NLW), AAs, and Latinos (see Tables 1 and 2, ranked by age-adjusted mortality rates in NLW; refs. 18, 19). We then estimated the disparity ratio (DR, the ratio of age-adjusted mortality between NLW and minorities) for men and women. These analyses identified 11 different priority cancer health disparity malignancies, including nine cancer types in women and eight cancer types in men (Tables 1 and 2). In Table 3, we show the number of PDX models available in PDXNet derived from NLW, AA, and Latino patients (based on self-reported race and ethnicity) from the priority cancer health disparity malignancies shown in Tables 1 and 2. Our results show that of the 10 malignancies, only breast cancer has a relatively large number of race/ethnicity-appropriate models for both AAs and Latinos. Unfortunately, for the majority of cancers, the number of available models is dismal (with no models available for many cancer types), indicating that more work needs to be done to address this important cancer health disparity research gap.

**Table 1:** Top 10 female age-adjusted mortality (per 100,000) and 5-year number of deaths (count) in NLW, AAs, and Latinos. Priority cancer health disparity malignancies have disparity ratio, DR, >1). Incidence-based mortality data from SEER (18 Registries, November 2019 Sub, 2000–2017)

	Non-Latino Whites			African Americans				Latinos			
Site	Rank	Mortality rate	Count	Rank	Mortality rate	Count	DR	Rank	Mortality rate	Count	DR
Breast	1	44.35	84,996	1	53.18	14,211	1.20	1	28.58	9,925	0.64
Lung and bronchus	2	38.73	71,424	2	36	9,726	0.93	2	16.83	5,451	0.43
Colon and rectum	3	19.92	39,257	3	25.82	6,793	1.30	3	14.77	4,932	0.74
Corpus and uterus	4	9.39	17,893	4	13.34	3,657	1.42	5	7.6	2,652	0.81
Pancreas	5	8.9	16,697	5	11.87	3,200	1.33	4	8.46	2,811	0.95
Lymphoma	6	8.34	16,126	7	6.63	1,735	0.79	6	7.5	2,426	0.90
Ovary	7	7.47	13,569	8	6.27	1,730	0.84	7	5.99	2,158	0.80
Leukemia	8	5.81	10,884	10	5.39	1,409	0.93	10	4.49	1,636	0.77
Skin^a^	9	5.55	10,881	28	0.63	162	0.11	20	1.61	535	0.29
Urinary bladder	10	5.16	10,443	13	4.31	1,091	0.84	15	2.54	781	0.49
Kidney and renal pelvis	11	4.52	8,619	9	5.48	1,426	1.21	11	4.41	1,462	0.98
Myeloma	14	2.89	5,448	6	7.68	2,002	2.66	13	3.16	1,019	1.09
Liver and bile duct	15	2.86	5,265	14	4.23	1,217	1.48	8	5.97	2,004	2.09
Cervix uteri	16	2.73	4,208	11	5.18	1,427	1.90	12	3.84	1,497	1.41
Stomach	17	2.29	4,284	12	5.04	1,317	2.20	9	5.52	1,959	2.41

a Excluding Basal and Squamous.

**Table 2:** Top 10 male age-adjusted mortality rates (per 100,000) and 5-year number of deaths (count) in NLWs, AAs, and Latinos. Priority cancer health disparity malignancies have disparity ratio, DR, >1). Incidence-based mortality data from SEER (18 Registries, November 2019 Sub, 2000–2017)

	Non-Latino Whites			African Americans				Latinos			
Site	Rank	Mortality rate	Count	Rank	Mortality rate	Count	DR	Rank	Mortality rate	Count	DR
Prostate	1	66.63	92,170	1	125.6	19,303	1.89	1	51.2	10,477	0.77
Lung and bronchus	2	49.97	75,245	2	60.7	11,920	1.21	2	25.15	6,157	0.50
Colon and rectum	3	28.92	41,546	3	37.88	6,935	1.31	3	23.56	6,060	0.81
Urinary bladder	4	24.09	33,566	6	12.68	1,990	0.53	6	10.37	2,189	0.43
Skin^a^	5	15.79	22,136	21	1.24	212	0.08	16	2.52	629	0.16
Lymphoma	6	14.09	20,030	8	10.92	2,181	0.78	5	11.55	3,041	0.82
Pancreas	7	11.46	17,479	5	13.78	2,840	1.20	7	9.6	2,669	0.84
Leukemia	8	10.91	15,398	11	8.24	1,529	0.76	10	7.13	2,143	0.65
Kidney and renal pelvis	9	10.39	15,294	7	12.31	2,321	1.18	8	9.53	2,566	0.92
Liver and bile duct	10	8.35	13,623	4	13.88	3,375	1.66	4	15.79	4,955	1.89
Stomach	13	5.61	8,325	10	10.29	1,956	1.83	9	9.42	2,624	1.68
Myeloma	14	5.14	7,496	9	10.54	1,903	2.05	11	4.96	1,239	0.96

^a^Excluding Basaling and Squamous.

**Table 3:** Number of available models for priority cancer health disparity malignancies

		Average annual deaths (2013–2017)/available sex-matched models		
Cancer type	Disproportionally affected group	Non-Latino Whites	African Americans	Latinos
Lung and bronchus	African American, men	29,334/44	4,329/2	2,322/0
Prostate	African American, men	18,434/7	3,861/0	2,095/0
Breast	African Americans, women	16,999/25	2,842/13	1,985/22
Colon and rectum	African Americans, women and men	16,161/109	2,746/4	2,198/3
Pancreas	African Americans, women and men	6,835/27	1,208/4	1,096/0
Liver and Intrahepatic bile duct	African Americans and Latinos, women and men	3,778/3	918/0	1,392/1
Uterus	African Americans, women	3,579/37	731/3	530/2
Kidney and renal pelvis	Latinos, men	3,059/22	464/1	513/0
Multiple myeloma	African Americans, women and men	2,589/0	781/0	1,096/0
Stomach	African Americans and Latinos, women and men	2,522/1	655/1	917/1
Cervix	African Americans and Latinos, women	842/5	285/0	299/1

## Discussion

In this study, we developed a cloud-based genetic ancestry pipeline, which we used to assess the genetic ancestry diversity of PDXnet models. We found that our genetic ancestry estimates had a high correspondence with self-reported race and ethnicity and thus are useful for comparing the model diversity to cancer health disparities data. Patients with non-European genetic ancestry are highly under-represented in the PDXNet models, which reflects similar disparities in other cancer resources (2). Recent efforts of the PDXNet have yielded an increase in 15 models of breast cancers likely derived from patients with African genetic ancestry and 23 models likely derived from Latin American genetic ancestry, representing a 3-fold and 23-fold increase in models above those previously available, respectively. Furthermore, with the addition of two minority and disparity-focused centers in PDXNet in late 2018, several models from minority patients will soon be deposited in the PDMR. Yet, as our power analyses showed, there are still far fewer models than would be required to conduct a study with sufficient power to identify models with genetic variants with biologically relevant effects on the cancer health disparities between demographic groups. This highlights the critical need for further investment in model development to help reach health equity goals.

Health disparities are complex and involve the interaction of many factors, including structural inequities, social determinants of health, cultural factors, and variance in exposure to environmental harms (3–5). Our focus on genetics in this study is motivated by our belief that precision medicine holds great promise for improving patient outcomes and that this promise should be realized equitably. Cancer evolution leverages the germline and somatic background of patients in which tumorigenesis occurs and the progression of cancer depends on the interactions between somatic mutations and the normal tissue in the microenvironment on which it grows (20). It is therefore important to understand the diversity of genetic backgrounds in which cancers evolve to identify relevant biomarkers that could help to address treatment disparities. We want to emphasize that human genetic diversity is complex, there is large variance within human populations and we do not seek to naively assign risk factors for cancer incidence to broad, biologically dubious categories (21–23). Rather, we hope this work will help to motivate and facilitate an understanding of whether precision medicine approaches have the potential to help address current cancer health disparities by increasing the number and diversity of models available to identify whether and how segregating germline and somatic variants can help explain those disparities.

Categorization of models based on continental ancestry has several limitations. The concept of continental ancestry is premised on the concept of continental races, the biological and biomedical relevance of which is debated and controversial (21, 24). The history of human migration and gene flow is complex and cannot be circumscribed by continental borders (22, 23). Additionally, analyses based on the categorization of individuals by self-reported race or ancestry likely elide the complex interactions of demography, the environment, and socioeconomic factors (3, 4, 23). Our goal in this study was to help motivate the critical need for cancer models that better reflect the diversity of human genetic variation in order to help achieve equity in precision medicine. In this way, we seek to better understand the extent to which genetic variants that segregate among demographic groups impact cancer health disparities.

In a recent commentary, we argued for the need to diversify patient-derived models and the implications of the limited diversity of such models to advance precision medicine in minority populations (2). In Table 3, we showed that there remain large gaps for models available for model development from cancers with high burden in minorities; we referred to these cancers above as “priority cancer health disparity malignancies.” For stomach tumors, a malignancy that disproportionally affects both minority groups, only one appropriate PDX exists for AA and another one for Latinos. For liver cancer, another malignancy with a high burden in both groups, there are no race/ethnicity-appropriate models for AA and only one for Latinos in PDXNet. Kidney tumors also lack race/ethnic-appropriate models from Latinos. The number of models for malignancies with high burden in AA is also dismal, with no race/ethnicity-appropriate models for multiple myeloma or prostate cancer, four for pancreatic cancer, three for endometrial cancer, four for colorectal cancer, and two for lung cancer (Table 3). Only breast cancer is moderately represented for AAs, with 19 models from this minority group currently available in PDXNet. While rare cancers in NLW, such as multiple myeloma and stomach, liver, and kidney tumors, also have low numbers for this majority racial group, the disparities for common NLW cancers are striking. For example, there is only one colorectal cancer PDX from Latinos for every ∼36 NLW models. For each AA colorectal cancer PDX, there are 27 NLW models. The number of pancreas and lung cancer PDXs are also 7-fold and 22-fold higher for NLW than for AA, respectively. These analyses, therefore, not only identify disparities in existing models but also highlight that developing more diverse preclinical models is needed to address cancer mortality disparities through race/ethnicity-appropriate preclinical and translational research. Given the disproportionally high burden caused by liver and stomach tumors in AA and Latinos, by renal tumors in Latinos, by colorectal, pancreas, lung, prostate, breast, and uterine tumors, and multiple myeloma in AA, we suggest that they should represent priorities for model development in PDXNet and similar initiatives.

While acknowledging that PDX development requires specialized infrastructure and is time- and resource-intensive, we believe that a number of approaches can be taken to increase the number and diversity of models in the future. One approach is to support such efforts in cancer centers in historically under-served communities. In PDXNet, for example, the two centers contributing the most diverse models included the Baylor College of Medicine Cancer Center, which serves the largest safety net hospital in Houston, and the UC Davis Cancer Center, which recruits patients throughout the University of California System Comprehensive Cancer Center, which accept patients with MediCal (California’s Medicaid program) insurance, many of whom are ethnic/racial minorities. Another related approach is to increase model diversity to support such efforts in racially/ethnically diverse states. It is unsurprising that the centers contributing the largest number of models from ethnic/racial minorities were in California and Texas, two of the most diverse states in the nation. A third approach is to support collaborations and researchers based in cancer hospitals and research institutions in Latin America and Africa. Having the ability to make models from African and Latin American patients should also be encouraged as many of these individuals share genetic ancestry with U.S. minority populations.

In conclusion, we developed and implemented a pipeline for genetic ancestry evaluations in publicly available patient-derived models and highlight the fact that most of them have a predominantly European genetic ancestry. We estimated that to understand biological differences and responses to therapy between different genetic ancestries, hundreds of models are needed, highlighting the need to diversify the models. Furthermore, using self-reported race/ethnicity information available in ∼63% of the models and cancer health disparities data in the two largest U.S. minority populations, we showed that there very few or, in many cases, no models to develop therapies for the cancer types with the highest burden in minority patients. While ongoing efforts promise to diversify these available models, we encourage funders to support additional efforts aimed at developing and characterizing new models that equitable help realize the promise of cancer precision medicine to all Americans.

## Supplementary Material

Figure S1 Figure S1 shows the Clustering of 1,990 non-admixed reference samples and 2,387 admixed samples(brown) individuals based on the first three principal components. Colors represent the continental ances

Figure S2 Figure S2 shows Diversity of genetic ancestry estimates from PDXNet breast cancer models. A: Inferred genetic ancestry for 115 breast cancer models in PDXnet. B: Top two categories for 26 "MIXED" samp

Figure S3 Figure S3 Shows the Power to detect a driver mutation that is absent in EUR but present in a non-EUR category at varying low frequencies.

Figure S4 Figure S4 Shows the Power to identify at least 5 patients with a known driver mutation that is present in populations at varying low frequencies.

Supplementary Data The full list of collaborators for PDXNet Consortium and Supplementary Data and Methods

Table S1 Populations of origin for unrelated individuals from the 1000 Genomes Phase III, GenomeAsia, and INMEGEN data used as reference populations to design a new SNPweights panel.

Table S2 PDXnet metadata for samples for which genetic ancestry was estimated and samples were assigned into African (AFR), European (EUR), East Asian (EAS), American (AMR), and South Asian (SAS) continental ancestry.

Table S3 1000Genomes K=10 Admixture Estimates downloaded from ftp://ftp.1000genomes.ebi.ac.uk/vol1/ftp/release/20130502/supporting/admixture_files/ALL.wgs.phase3_shapeit2_filtered.20141217.maf0.05.10.Q. Super-population designations were added to the partitions by sorting each partition and determining the corresponding designation for each partition.

Table S4 Ancestral estimates 1000Genomes individuals (n=929) not used in reference panel generation. Estimates calculated from 1000Genomes K=10 admixture data, SNPWeights Panel, and the differences between these estimates for each individual.

Table S5 Mean and Stdev of differences for 5 continental ancestral estimations between SNPWeights Panel and 1000Genomes K=10 Admixture Estimates by individual's super-population designation.
