# Supplementary figures and images for: Development and Application of Genetic Ancestry Reconstruction Methods to Study Diversity of Patient-Derived Models in the NCI PDXNet Consortium
Source: Cancer Res Commun. 2024 Aug 16;4(8):2147–52. doi: 10.1158/2767-9764.CRC-23-0417 (PMC11328913; doi:10.1158/2767-9764.CRC-23-0417)

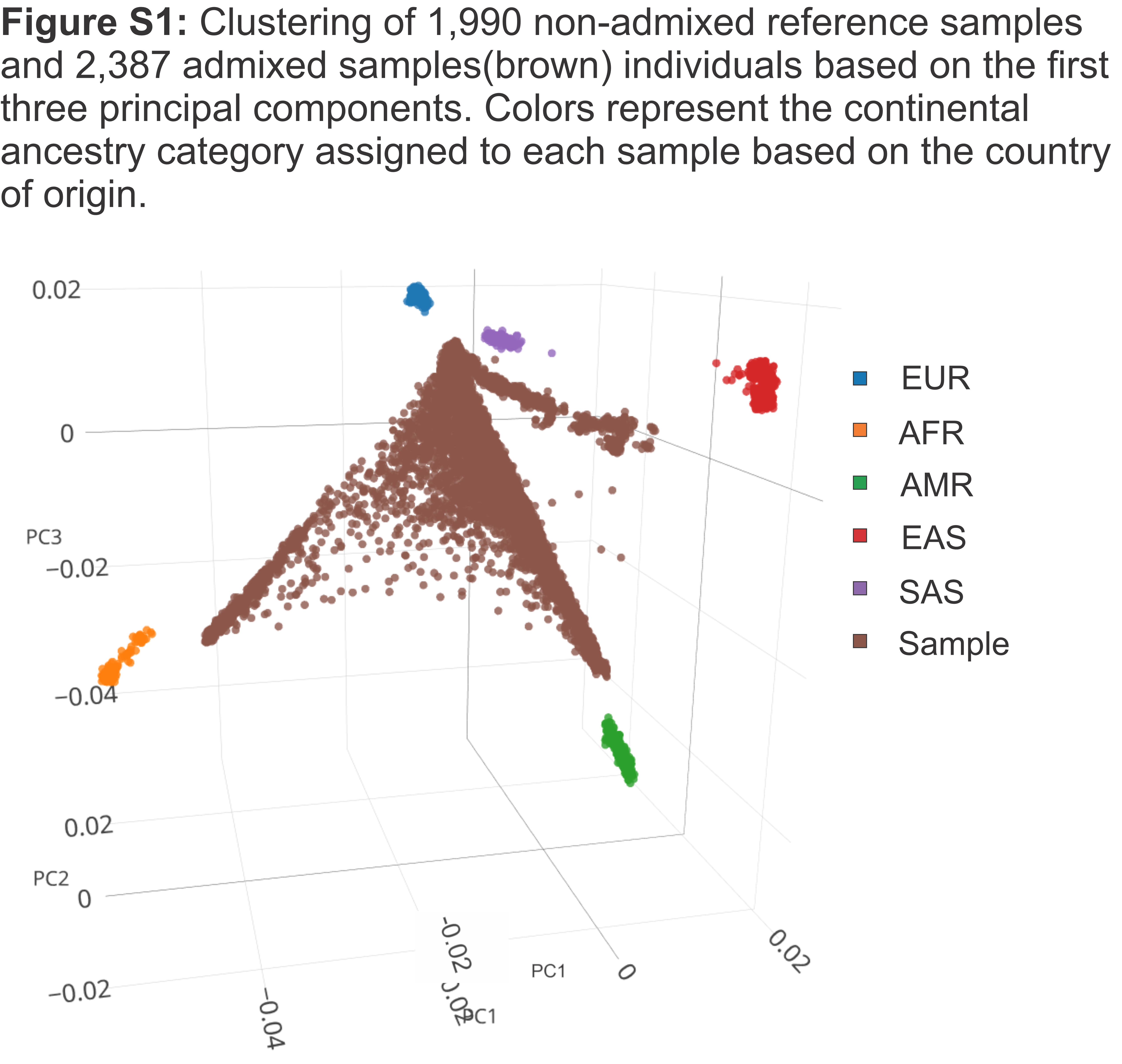

Supplement: Figure S1 — shows the Clustering of 1,990 non-admixed reference samples and 2,387 admixed samples(brown) individuals based on the first three principal components. Colors represent the continental ances [file crc-23-0417_figure_s1_suppsf1.png]

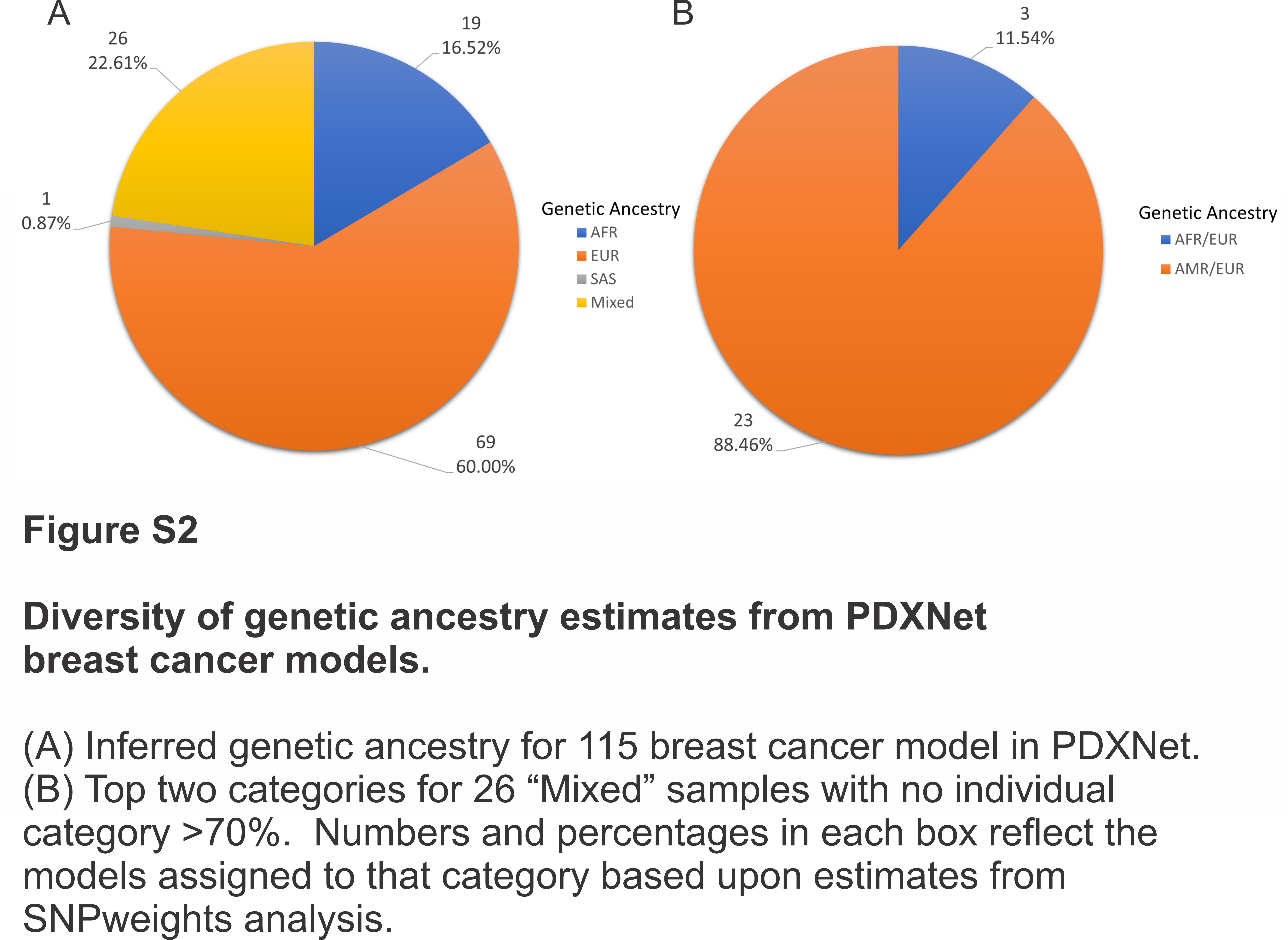

Supplement: Figure S2 — shows Diversity of genetic ancestry estimates from PDXNet breast cancer models. A: Inferred genetic ancestry for 115 breast cancer models in PDXnet. B: Top two categories for 26 "MIXED" samp [file crc-23-0417_figure_s2_suppsf2.png]

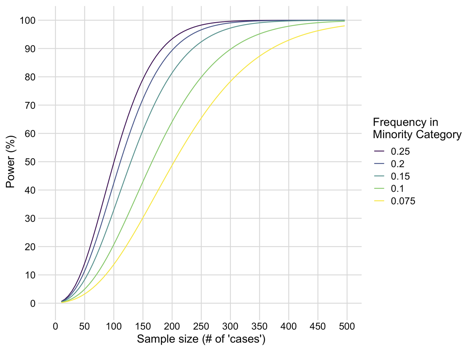

Supplement: Figure S3 — Shows the Power to detect a driver mutation that is absent in EUR but present in a non-EUR category at varying low frequencies. [file crc-23-0417_figure_s3_suppsf3.png]

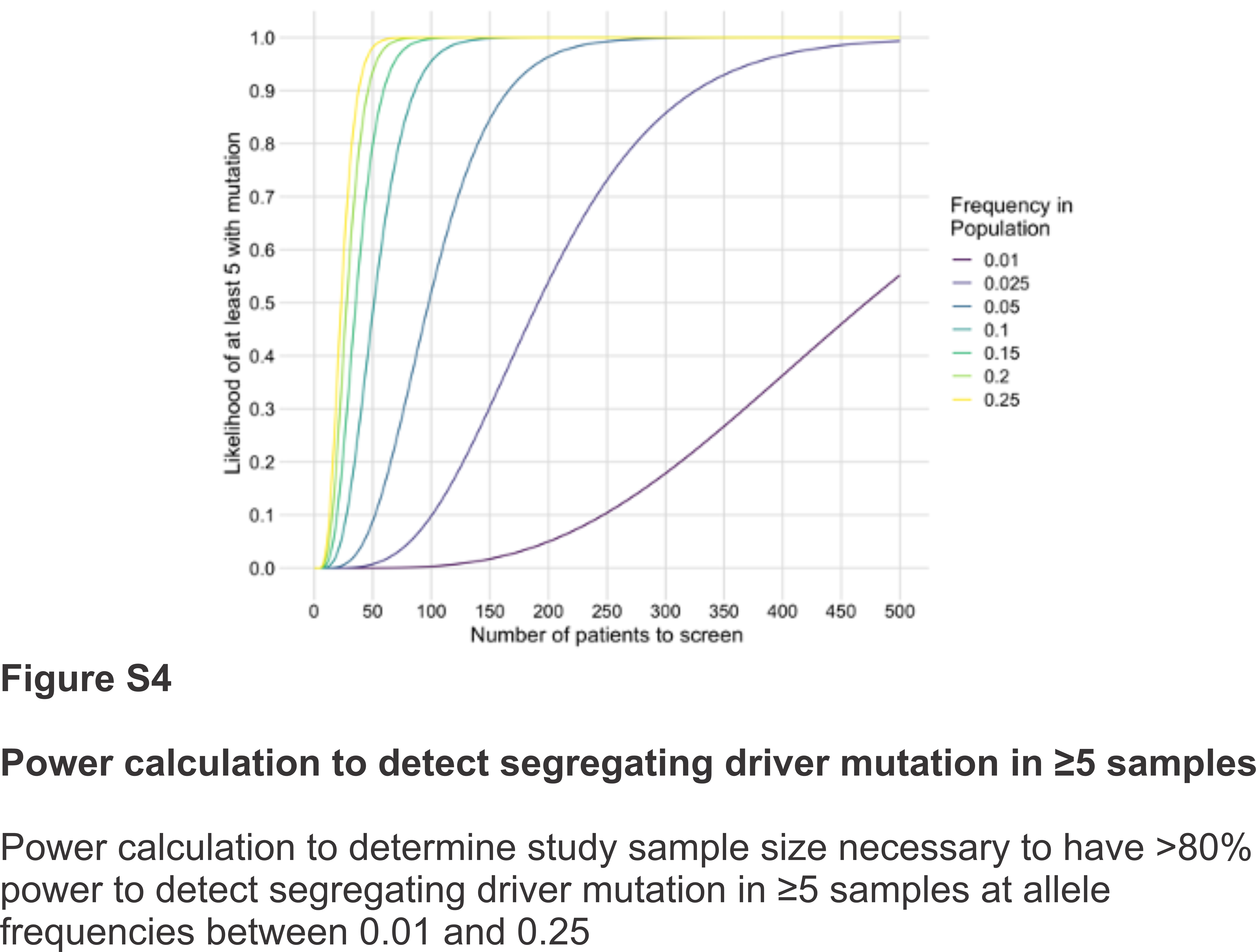

Supplement: Figure S4 — Shows the Power to identify at least 5 patients with a known driver mutation that is present in populations at varying low frequencies. [file crc-23-0417_figure_s4_suppsf4.png]
