# Supplementary material for: Development and Application of Genetic Ancestry Reconstruction Methods to Study Diversity of Patient-Derived Models in the NCI PDXNet Consortium
Source: Cancer Res Commun. 2024 Aug 16;4(8):2147–52. doi: 10.1158/2767-9764.CRC-23-0417 (PMC11328913; doi:10.1158/2767-9764.CRC-23-0417)
Supplement: Supplementary Data — The full list of collaborators for PDXNet Consortium and Supplementary Data and Methods [file crc-23-0417_supplementary_data_suppsd.docx]

# Supplementary Data

The full list of collaborators for PDXNet Consortium is shown below:

Alana Welm, Huntsman Cancer Institute/Baylor College Of Medicine

Bryan Welm, Huntsman Cancer Institute/Baylor College Of Medicine

Michael T. Lewis, Huntsman Cancer Institute/Baylor College Of Medicine

Matthew Bailey, Huntsman Cancer Institute/Baylor College Of Medicine

Emilio Cortes-Sanchez, Huntsman Cancer Institute/Baylor College Of Medicine

Sandra Scherer, Huntsman Cancer Institute/Baylor College Of Medicine

Chieh-Hsiang Yang, Huntsman Cancer Institute/Baylor College Of Medicine

Andrew Butterfield, Huntsman Cancer Institute/Baylor College Of Medicine

Zhengtao Chu, Huntsman Cancer Institute/Baylor College Of Medicine

Maihi Fujita, Huntsman Cancer Institute/Baylor College Of Medicine

Ling Zhao, Huntsman Cancer Institute/Baylor College Of Medicine

Lacey E. Dobrolecki, Huntsman Cancer Institute/Baylor College Of Medicine

Heidi Dowst, Huntsman Cancer Institute/Baylor College Of Medicine

Jack Roth, MD Anderson Cancer Center

Funda Meric-Bernstam, MD Anderson Cancer Center

Argun Akcakanat, MD Anderson Cancer Center

Gao Boning, MD Anderson Cancer Center

Kurt Evans, MD Anderson Cancer Center

Bingliang Fang, MD Anderson Cancer Center

Don Gibbons, MD Anderson Cancer Center

Vanessa Jensen, MD Anderson Cancer Center

Dara Keener, MD Anderson Cancer Center

Michael Kim, MD Anderson Cancer Center

Scott Kopetz, MD Anderson Cancer Center

Mourad Majidi, MD Anderson Cancer Center

David Menter, MD Anderson Cancer Center

John Minna, MD Anderson Cancer Center

Hyunsil Park, MD Anderson Cancer Center

Coya Tapia, MD Anderson Cancer Center

Brenda Timmons, MD Anderson Cancer Center

Jing Wang, MD Anderson Cancer Center

Shannon Westin, MD Anderson Cancer Center

Timothy Yap, MD Anderson Cancer Center

Jianhua Zhang, MD Anderson Cancer Center

Ran Zhang, MD Anderson Cancer Center

Xiaoshan Zhang, MD Anderson Cancer Center

Min Jin Ha, MD Anderson Cancer Center

Huiqin Chen, MD Anderson Cancer Center

Yuanxin Xi, MD Anderson Cancer Center

Dali Li, MD Anderson Cancer Center

Luc Girard, MD Anderson Cancer Center

Xiaofeng Zheng, MD Anderson Cancer Center

Erkan Yucan, MD Anderson Cancer Center

Christopher D. Lanier, MD Anderson Cancer Center

Turcin Saridogan, MD Anderson Cancer Center

Bryce P. Kirby, MD Anderson Cancer Center

Bingbing Dai, MD Anderson Cancer Center

Jitesh Augustine, MD Anderson Cancer Center

Ismail Meraz, MD Anderson Cancer Center

Alexey Sorokin, MD Anderson Cancer Center

Fei Yang, MD Anderson Cancer Center

Kelly Gale, MD Anderson Cancer Center

Stephen Scott, MD Anderson Cancer Center

Yi Xu, MD Anderson Cancer Center

Jeffrey H. Chuang, PDX Data Commons and Coordination Center

Brandi Davis-Dusenbery, PDX Data Commons and Coordination Center

Carol J. Bult, PDX Data Commons and Coordination Center

Peter N. Robinson, PDX Data Commons and Coordination Center

Sergii Domanskyi, PDX Data Commons and Coordination Center

Michael W. Lloyd, PDX Data Commons and Coordination Center

Steven B. Neuhauser, PDX Data Commons and Coordination Center

Jill Rubinstein, PDX Data Commons and Coordination Center

Brian J. Sanderson, PDX Data Commons and Coordination Center

Anuj Srivastava, PDX Data Commons and Coordination Center

Xing Yi Woo, PDX Data Commons and Coordination Center

Brian S. White, PDX Data Commons and Coordination Center

Saul Acevedo, PDX Data Commons and Coordination Center

Vicki Chin, PDX Data Commons and Coordination Center

Dennis A. Dean, II, PDX Data Commons and Coordination Center

John DiGiovanna, PDX Data Commons and Coordination Center

Soner Koc, PDX Data Commons and Coordination Center

Phillip Webster, PDX Data Commons and Coordination Center

Jelena Randjelovic, PDX Data Commons and Coordination Center

Meenhard Herlyn, Wistar & MD Anderson Cancer Center

Mike Davies, Wistar & MD Anderson Cancer Center

Dylan Fingerman, Wistar & MD Anderson Cancer Center

Andrew Kossenkov, Wistar & MD Anderson Cancer Center

Qin Liu, Wistar & MD Anderson Cancer Center

Kate Nathanson, Wistar & MD Anderson Cancer Center

Vito Rebecca, Wistar & MD Anderson Cancer Center

Rajasekharan Somasundaram, Wistar & MD Anderson Cancer Center

Mike Tetzlaff, Wistar & MD Anderson Cancer Center

Jayamanna Wickramasinghe, Wistar & MD Anderson Cancer Center

Min Xiao, Wistar & MD Anderson Cancer Center

George Xu, Wistar & MD Anderson Cancer Center

Vashisht G. Yennu-Nanda, Wistar & MD Anderson Cancer Center

Haiyin Lin, Wistar & MD Anderson Cancer Center

Eric Ramirez-Salazar, Wistar & MD Anderson Cancer Center

Govindan Ramaswamy, Washington University in St. Louis

Li Ding, Washington University in St. Louis

Shunqiang Li, Washington University in St. Louis

Rebecca Aft, Washington University in St. Louis

Jessica Andrews, Washington University in St. Louis

Alicia Asaro, Washington University in St. Louis

Song Cao, Washington University in St. Louis

Feng Chen, Washington University in St. Louis

Sherri Davies, Washington University in St. Louis

John DiPersio, Washington University in St. Louis

Erin Dreskell, Washington University in St. Louis

Ryan Fields, Washington University in St. Louis

Steven Foltz, Washington University in St. Louis

Katherine Fuh, Washington University in St. Louis

Kian Lim, Washington University in St. Louis

Jinqin Luo, Washington University in St. Louis

Cynthia Ma, Washington University in St. Louis

Jay Mashl, Washington University in St. Louis

Mike McLellan, Washington University in St. Louis

Tina Primeau, Washington University in St. Louis

Fernanda Rodrigues, Washington University in St. Louis

Dawn Ross, Washington University in St. Louis

Roslynn Sims, Washington University in St. Louis

Hua Sun, Washington University in St. Louis

Brian VanTine, Washington University in St. Louis

Andrea Wang-Gillam, Washington University in St. Louis

Mike Wendl, Washington University in St. Louis

Cathy Wiggins, Washington University in St. Louis

Yige Wu, Washington University in St. Louis

Matt Wyczalkowski, Washington University in St. Louis

Lijun Yao, Washington University in St. Louis

Daniel Cui Zhou, Washington University in St. Louis

Julie Belmar, Washington University in St. Louis

Jeremy Hoog, Washington University in St. Louis

Reyka Jayasinghe, Washington University in St. Louis

Yize Li, Washington University in St. Louis

Kian-Huat Lim, Washington University in St. Louis

Chia-Kuei Mo, Washington University in St. Louis

Nadezhda Terekhanova, Washington University in St. Louis

Rose Tipton, Washington University in St. Louis

Jason Held, Washington University in St. Louis

Jacqueline Mudd, Washington University in St. Louis

Sidharth Puram, Washington University in St. Louis

Julie Schwarz, Washington University in St. Louis

Jose Zevallos, Washington University in St. Louis

Chong-xian Pan, University of California-Davis

Moon S. Chen, Jr, University of California-Davis

Luis Carvajal-Carmona, University of California-Davis

John Albeck, University of California-Davis

Katherine Chiu, University of California-Davis

Nicole Coggins, University of California-Davis

Edward Pugh, University of California-Davis m

David Rocke, University of California-Davis m

Susan Stewart, University of California-Davis m

Clifford Tepper, University of California-Davis m

April Vang, University of California-Davis m

Amanda Kirane, University of California-Davis m

May Cho, University of California-Davis

David Gandara, University of California-Davis

Jonathan Reiss, University of California-Davis

Tiffany Le, University of California-Davis

Ralph De Vere White, University of California-Davis

Lisa Brown, University of California-Davis

David Cooke, University of California-Davis

Marc Dall'Era, University of California-Davis

Sepideh Gholami, University of California-Davis

Luis Godoy, University of California-Davis

Rashmi Verma, University of California-Davis

Hongyong Zhang, University of California-Davis

Nicole Coggins, University of California-Davis

Paul Lott, University of California-Davis

Ana Estrada, University of California-Davis

Ted Toal, University of California-Davis

Alexa Morales, University of California-Davis

Guadalupe Polanco Echeverry, University of California-Davis

Sienna Rocha, University of California-Davis

David Segal, University of California-Davis

Ai-Hong Ma, University of California-Davis

Nicholas Mitsiades, Baylor College of Medicine

Salma Kaochar, Baylor College of Medicine

Matthew Ellis, Baylor College of Medicine

Michael Ittmann, Baylor College of Medicine

Susan Hilsenbeck, Baylor College of Medicine

Bert O'Malley, Baylor College of Medicine

Jeffrey Moscow, National Cancer Institute

Tiffany Wallace, National Cancer Institute

James Doroshow, National Cancer Institute

Xiaowei Xu, National Cancer Institute

Li Chen, National Cancer Institute

Rajesh Patidar, National Cancer Institute

Yvonne Evrard, National Cancer Institute

# Supplemental Methods

Several algorithms exist to estimate ancestry from genetic data, which can be roughly categorized as model-based methods that rely on statistical inference based on, for example, admixture linkage disequilibrium, and distance-based methods that estimate ancestry using clustering, network theory, or graph theory.^1,2^ STRUCTURE was a trailblazing program that led to a proliferation of parametric methods that model and partition linkage disequilibrium due to admixture to ascertain the genetic structure of populations, or to identify the proportional contribution of different reference populations to the genetic makeup of individuals.^3,4^ Although this type of model-based method can provide robust estimates of ancestry, they often require detailed population or pedigree information that may not be readily available from protected or anonymized patient data, and they also tend to have long run-times and detailed hands-on analyses that make them less tractable for pipeline development.^5^ Alternatively, distance-based approaches use methods such as principal component analysis (PCA)^6,7^ to assign genetic ancestry by projecting samples into high-dimensional space and comparing their positions relative to data from reference populations, which is a much less computationally intensive task than most model-based methods.

## SNPweights Panel Design

SNPweights v 2.1 includes several weight panels that differ in the representation of continental reference populations.^8^ However, in our benchmark testing of published SNPweights models, we found that the “NA” panel, which includes reference data from African, East Asian, European, and Indigenous American populations, yielded ancestry estimates that were not concordant with ADMIXTURE estimates using 1000 Genomes and Indigenous American references. We designed a new reference panel for the program SNPweights to improve the classification of individuals from ancestral backgrounds that originated in the Americas and South Asia. We used as a reference set human genetic data that were downloaded from the 1000 Genomes Project Phase III,^9^ GenomeAsia 100K^10^, and INMEGEN^11^ (Table S1), with data imputed using the TOPmed Imputation Server.^12^ We estimated continental genetic ancestral fractions for each sample as fractions of five continental ancestral categories, which include European (EUR), African (AFR), Indigenous American (AMR), East Asia (EAS), and South Asian (SAS)^13^.

Reference data were converted from the Genome Reference Consortium (GRC) GRCh37 build of the human genome^14^ to the GRCh38 build^15^ using UCSC LiftOver.^16,17^ The raw data consisted of 4,471,291 SNPs identified in 11,448 individuals. A principal component analysis was performed on the filtered data using SMARTPCA in the EIGENSOFT package v. 7.2.0^6,7^ to identify 1,990 individuals with little to no admixture, which formed tight clusters at the termini of the ancestral axes (238 African, 348 Indigenous American, 408 European, 766 East Asian, 230 South Asian; Fig. S1). Markers were filtered for minor allele frequency ≤ 5%, Hardy-Weinberg equilibrium P ≤ 0.0005, independent pairwise linkage disequilibrium pruning using a 50Kbp window a step size of 5 markers and r^2^ threshold of ≥ 0.5, and exclusion of high LD regions, leaving 264,153 SNPs.

The filtered data were converted into EIGENSTRAT format using convertf in the EIGENSOFT package v. 7.2.0.^6,7^ A principal component analysis was performed on the filtered data using SMARTPCA in the EIGENSOFT package v. 7.2.0. Finally, the weighting factors for ancestry inference were extracted from the PCA results using the program calc_snpwt.py from the SNPweights package v 2.1.

This model was validated and benchmarked using 2,387 admixed individuals with ADMIXTURE v1.3.0^18^ in both supervised and unsupervised modes. In our assessment of the original SNPweights NA panel’s estimates of Asian ancestry, individuals identified by 1000 Genomes as SAS differed on average by 0.5507 (SD:0.0910) in comparison to ADMIXTURE supervised. Among the same 1000 Genomes individuals identified as AMR and SAS, the new SNPweights ancestral estimates differed from ADMIXTURE supervised by mean of 0.0231 (SD: 0.0167) and 0.0369 (SD: 0.0400), respectively.

## Ancestry Estimation

To assess the diversity of genetic backgrounds represented by the PDX models in PDXNet, we estimated genetic ancestry using the Binary Alignment Map (BAM) files that were prepared as part of the standard intake process for whole exome data on the CGC.^19^ Genotypes were estimated using the programs bcftools mpileup with the parameters -I -q 30 -Q 20 and bcftools call with the parameter -m in the bcftools package v. 1.9.^20^ These genotypes were then filtered using bcftools filter with the parameters -g3 -G10 -e’%QUAL<20 || (RPB<0.1 && %QUAL<25) || (AC<2 && %QUAL<25) || INFO/DP>70 || INFO/DP<4’. The filtered genotypes were annotated with SNP IDs from dbSNP build 151^19^ using bcftools annotate. The annotated genotypes were converted into EIGENSTRAT format using the program vcf2eigenstrat.py in the Genetic Data Conversion package.^22^ Finally, genetic ancestry proportions were estimated using the program inferanc in the SNPweights package v 2.1. Samples were then categorized into the ancestry group for which the proportion was greater than 0.7. When no category scored greater than 0.7 the samples were labeled MIXED, and were categorized based on the top two ancestry groups for those samples.

## Power Analyses

To determine the power to detect driver mutations that differ in frequency between demographic groups, we performed a power analysis using the Power for Genetic Association Analyses (PGA) package.^23^ Statistical power was modeled for a dominant mode of inheritance, assuming that markers and causative SNPs are in complete linkage (LD = 1), and that there were 200 effective degrees of freedom, which would account for 200 comparisons due to multiple genetic markers. The causative mutation was modeled to be absent in one group, and present at varying low frequencies of 0.075, 0.1, 0.15, 0.2, 0.25.

We were also interested to determine the sampling effort necessary to develop new PDX models for a newly discovered driver mutation that is segregating at a known frequency in a minority population. We modeled the sampling effort necessary to identify at least five individuals with the driver mutation using the hypergeometric distribution across varying frequencies from 0.01 to 0.25 using the function phyper in R.^24^ Plots from both power analyses were prepared using the package ggplot2 in R.^25^

# *Cancer Health Disparities Data Analyses*

We used data from the NCI Surveillance, Epidemiology, and End Results program from 18 population-based cancer registries (SEER18) from 2013-2017 to identify the top 10 causes of cancer mortality in African Americans (AA) and Latinos, the two largest US minority populations, and in Non-Latino Whites (NLW). As PDXs are primarily used for pre-clinical studies to develop new therapies, we focused on analyses in cancer mortality disparities. In these analyses, once the top ten causes of cancer mortality in men and women from each of the three racial/ethnic groups were identified, we estimated the fold difference in mortality between minority populations and NLW, which we termed the disparity ratio. Cancers for which mortality rates ranked exclusively high in minorities or that have disparity ratios > 1 were then designated as “priority malignancies” for model development. We then queried the PDXNet dataset to identify the number of ethnic/race appropriate models for such priority malignancies and estimated the number of models available, based on self-reported race and ethnicity.

# *Data Accessibility*

The new SNPweights panel will be made publicly available upon acceptance of this manuscript. The ancestry estimation pipeline is implemented in the Common Workflow Language and will be made available in the public applications gallery of the Cancer Genomics Cloud upon acceptance of this manuscript.

**References**

1., Padhukasahasram B. Inferring ancestry from population genomic data and its applications. *Front Genet*. 2014;5:204. doi:10.3389/fgene.2014.00204

2., Jin Y, Schaffer AA, Feolo M, Holmes JB, Kattman BL. GRAF-pop: A Fast Distance-Based Method To Infer Subject Ancestry from Multiple Genotype Datasets Without Principal Components Analysis. *G3 Bethesda Md*. 2019;9(8):2447-2461. doi:10.1534/g3.118.200925

3., Pritchard JK, Stephens M, Donnelly P. Inference of population structure using multilocus genotype data. *Genetics*. 2000;155(2):945-959.

4., Porras-Hurtado L, Ruiz Y, Santos C, Phillips C, Carracedo A, Lareu MV. An overview of STRUCTURE: applications, parameter settings, and supporting software. *Front Genet*. 2013;4:98. doi:10.3389/fgene.2013.00098

5., Geza E, Mugo J, Mulder NJ, Wonkam A, Chimusa ER, Mazandu GK. A comprehensive survey of models for dissecting local ancestry deconvolution in human genome. *Brief Bioinform*. 2019;20(5):1709-1724. doi:10.1093/bib/bby044

6., Price AL, Patterson NJ, Plenge RM, Weinblatt ME, Shadick NA, Reich D. Principal components analysis corrects for stratification in genome-wide association studies. *Nat Genet*. 2006;38(8):904-909. doi:10.1038/ng1847

7., Patterson N, Price AL, Reich D. Population structure and eigenanalysis. *PLoS Genet*. 2006;2(12):e190. doi:10.1371/journal.pgen.0020190

8., Chen CY, Pollack S, Hunter DJ, Hirschhorn JN, Kraft P, Price AL. Improved ancestry inference using weights from external reference panels. *Bioinformatics*. 2013;29(11):1399-1406. doi:10.1093/bioinformatics/btt144

9., Auton A, Brooks LD, Durbin RM, et al. A global reference for human genetic variation. *Nature*. 2015;526(7571):68-74. doi:10.1038/nature15393

10., GenomeAsia100K Consortium, Wall JD, Stawiski EW, et al. The GenomeAsia 100K Project enables genetic discoveries across Asia. *Nature*. 2019;576(7785):106-111. doi:10.1038/s41586-019-1793-z

11., Moreno-Estrada A, Gignoux CR, Fernández-López JC, et al. The genetics of Mexico recapitulates Native American substructure and affects biomedical traits. *Science*. 2014;344(6189):1280-1285. doi:10.1126/science.1251688

12., Das S, Forer L, Schönherr S, et al. Next-generation genotype imputation service and methods. *Nat Genet*. 2016;48(10):1284-1287. doi:10.1038/ng.3656

13., Constantinescu AE, Mitchell RE, Zheng J, et al. A framework for research into continental ancestry groups of the UK Biobank. *Hum Genomics*. 2022;16(1):3. doi:10.1186/s40246-022-00380-5

14., Church DM, Schneider VA, Graves T, et al. Modernizing reference genome assemblies. *PLoS Biol*. 2011;9(7):e1001091. doi:10.1371/journal.pbio.1001091

15., Schneider VA, Graves-Lindsay T, Howe K, et al. Evaluation of GRCh38 and de novo haploid genome assemblies demonstrates the enduring quality of the reference assembly. *Genome Res*. 2017;27(5):849-864. doi:10.1101/gr.213611.116

16., Hinrichs AS, Karolchik D, Baertsch R, et al. The UCSC Genome Browser Database: update 2006. *Nucleic Acids Res*. 2006;34(Database issue):D590-598. doi:10.1093/nar/gkj144

17., Haeussler M, Zweig AS, Tyner C, et al. The UCSC Genome Browser database: 2019 update. *Nucleic Acids Res*. 2019;47(D1):D853-D858. doi:10.1093/nar/gky1095

18., Alexander DH, Novembre J, Lange K. Fast model-based estimation of ancestry in unrelated individuals. *Genome Res*. 2009;19(9):1655-1664. doi:10.1101/gr.094052.109

19., Lau JW, Lehnert E, Sethi A, et al. The Cancer Genomics Cloud: Collaborative, Reproducible, and Democratized—A New Paradigm in Large-Scale Computational Research. *Cancer Res*. 2017;77(21):e3-e6. doi:10.1158/0008-5472.CAN-17-0387

20., Danecek P, Bonfield JK, Liddle J, et al. Twelve years of SAMtools and BCFtools. *GigaScience*. 2021;10(2):giab008. doi:10.1093/gigascience/giab008

21., Sherry ST, Ward MH, Kholodov M, et al. dbSNP: the NCBI database of genetic variation. *Nucleic Acids Res*. 2001;29(1):308-311. doi:10.1093/nar/29.1.308

22., Mathieson I. gdc: genetic data conversion. Published 2018. Accessed October 29, 2020. https://github.com/mathii/gdc

23., Menashe I, Rosenberg PS, Chen BE. PGA: power calculator for case-control genetic association analyses. *BMC Genet*. 2008;9(1):36. doi:10.1186/1471-2156-9-36

24., R Core Team. *R: A Language and Environment for Statistical Computing*. R Foundation for Statistical Computing; 2020. https://www.R-project.org/

25., Wickham H. *Ggplot2: Elegant Graphics for Data Analysis*. Springer-Verlag New York; 2016. https://ggplot2.tidyverse.org
